# Assessment of soy‐based imports into the United States and associated foreign animal disease status

**DOI:** 10.1111/tbed.14284

**Published:** 2021-08-25

**Authors:** Allison K. Blomme, Cassandra K. Jones, Jordan T. Gebhardt, Jason C. Woodworth, Chad B. Paulk

**Affiliations:** ^1^ Department of Grain Science and Industry, College of Agriculture Kansas State University Manhattan Kansas USA; ^2^ Department of Animal Sciences and Industry, College of Agriculture Kansas State University Manhattan Kansas USA; ^3^ Department of Diagnostic Medicine/Pathobiology, College of Veterinary Medicine Kansas State University Manhattan Kansas USA

**Keywords:** foreign animal disease, soy oil cake, soy‐based imports, soybean meal, soybeans

## Abstract

Soy‐based products are known to pose a viable risk to U.S. swine herds because of their ability to harbour and transmit virus. This publication aimed to evaluate soy imports into the United States as a whole and from foreign animal disease positive (FAD‐positive) countries to determine which products are being imported in the highest quantities and observe potential trends in imports from FAD‐positive countries. Import data were accessed through the United States International Trade Commission website (USITC DataWeb) and summarized using R (version 4.0.2, R core team, Vienna, Austria). Twenty‐one different Harmonized Tariff Schedule (HTS) codes were queried to determine quantities (metric tonnes, MT) and breakdown of different soy product types being imported into the United States from 2015 to 2020. A total of 78 different countries exported soy products to the United States in 2019 and 2020 with top contributors being Canada (546,467 and 481,497 MT, respectively), India (397,858 and 430,621 MT, respectively) and Argentina (122,116 and 79,471 MT, respectively). Soy oilcake (582,273 MT) was imported in the largest quantities, followed by organic soybeans (270,194 MT) and soy oil (134,436 MT) for 2020. Of the 78 countries, 46 had cases of FAD reported through the World Organization for Animal Health (OIE) World Animal Health Information Database (WAHIS). Top exporters of soy products to the United States from FAD‐positive countries in 2019 and 2020 were India (397,858 and 430,621 MT, respectively), Argentina (122,116 MT in 2019) and Ukraine (40,293 and 56,392 MT, respectively). The risk of FAD introduction to the United States through soy imports can fluctuate based on where FAD outbreaks are occurring, shipping methods and end usage of products. A system to monitor these factors could help make future decisions about trade and risk of FAD introduction to U.S. swine herds.

## 1 INTRODUCTION

The ability of feed and feedstuffs to serve as a vector of disease for swine can have significant impacts on the U.S. swine industry. Feed has been associated with the U.S. porcine epidemic diarrhoea virus outbreak, which caused large death loss of nursery pigs and impacted pork supplies (Scott et al., 2016). Studies have shown that African swine fever virus (ASFV)‐contaminated feed has the ability to cause infection in pigs as well (Dee et al., 2014, 2018; Niederwerder et al., 2019; Scott et al., 2016). Several viruses have been shown to survive shipping models in a variety of feed ingredients including soy products, such as soybean meal and soy oilcake (Dee et al., 2018; Stoian et al., 2020). Several viruses included in this study such as ASFV, classical swine fever virus (CSFV), Ajueszky's disease (pseudorabies) and foot and mouth disease (FMDV) are foreign animal diseases (FADs) in the United States and of direct interest to the swine industry. Of these viruses, only pseudorabies and CSFV have been introduced to U.S. swine herds and eradicated (Brown & Bevins, 2018b; Haagmans et al., 1999). Vaccines exist for CSFV, FMDV and pseudorabies, but their introduction to the United States would still be detrimental to the industry (Beer et al., 2007; Haagmans et al, 1999; Knight‐Jones & Rushton, 2013). Of particular concern is ASFV because it has never been reported in the United States and does not currently have a vaccine against it. With this knowledge, it is critical to understand what feed ingredients are being imported to the United States and where they originated, so the risk level of FAD introduction can be evaluated. Of particular interest are soybean meal and soy oil because of their likelihood of being added to swine diets. Soy products have an increased ability to harbour viable virus when compared to other feed ingredients and organic soy is of particular interest because of the claim that most soy imports into the United States are organic (Dee et al., 2018; National Pork Producers Council, 2020). Some work has been done to prove an analytical approach to quantify soy imports into the United States but was focused only on ASFV‐positive countries and not on total imports of soy products regardless of FAD status (Patterson et al., 2021). Also, understanding the ports of entry (POEs) into the United States for these products is beneficial for understanding the shipping time and conditions that the majority of soy products experience. This information is useful when modelling shipping conditions or for product traceability. The objectives of this paper were (1) to evaluate annual soy imports into the United States by product type and determine the portion coming from countries with FAD, (2) evaluate POE into the United States and determine ports handling the largest volume of products and (3) track soy import trends with regard to imports from FAD‐positive countries.

## 2 METHODS

This work looked at import records from 2015 to 2020 with a particular focus on 2019 and 2020. Product classification, quantity, country of origin, POE and year were obtained through the International Trade Commission Harmonized Tariff Schedule website (DataWeb; https://dataweb.usitc.gov/). Product categories are identified by unique 10‐digit Harmonized Tariff Schedule (HTS) codes (Table 1). Twenty‐one HTS codes associated with soy products that have potential to be used in animal feed were used to query the database. Several products, such as lecithins or butter substitutes, were included that may end up in by‐products fed to animals. Data were exported to R (version 4.0.2, R core team, Vienna, Austria) where they were refined to total imports from each country by year and product type. Each HTS code was assigned a shortened description to improve data manipulation and reporting. Because of the low import rate of organic soy flour and meal; soy flour and meal; and soy flour and meal, not elsewhere specified or indicated (NESOI), these three HTS product categories were combined into one group in this report (Table 2). All soy oils, regardless of refinement level, were combined into one ‘soy oil’ category because of the low volume of imports in each subsection as well. POE provided information on the types of products coming from each country through the major ports in the United States. FAD‐positive countries were identified for each year and based on reported cases in any country that the World Organization for Animal Health (OIE) identified as having ASFV, CSFV, FMDV or Aujeszky's disease (pseudorabies) cases during that year in their World Animal Health Information Database (WAHIS; https://wahis.oie.int/#/dashboards/qd‐dashboard). Countries that had a case of ASFV, CSFV, FMDV or pseudorabies in their wild or domestic herds at any point in a year were considered positive and the rest were considered negative including countries with no report submitted. FAD status for each country by year was added to the import dataset and used to determine product types and amounts sourced from countries that experienced ASFV, CSFV, FMDV or pseudorabies in each year.

**TABLE 1:** Harmonized Tariff Schedule (HTS) codes utilized, their product descriptions and shortened names

HTS code	Product description	Short name
1201.90.0005	SOYBEAN SEEDS OF A KIND USED AS OIL STOCK, WHETHER OR NOT BROKEN	Soybean seeds
1201.90.0010	SOYBEANS, CERTIFIED ORGANIC, WHETHER OR NOT BROKEN, EXCEPT SEEDS OF A KIND USED FOR SOWING OR USED AS OIL STOCK	Organic soybeans
1201.90.0090	SOYBEANS, WHETHER OR NOT BROKEN, OTHER THAN CERTIFIED ORGANIC, NESOI	Non‐organic soybeans
1208.10.0000	FLOURS AND MEALS OF SOYBEANS	Soy flour and meal
1208.10.0010	FLOURS AND MEALS OF SOYBEANS, CERTIFIED ORGANIC	Organic soy flour and meal
1208.10.0090	FLOURS AND MEALS OF SOYBEANS, NESOI	Soy flour and meal, NESOI
1507.10.0000	SOYBEAN OIL AND ITS FRACTIONS, CRUDE, WHETHER OR NOT DEGUMMED	Crude oil
1507.90.4020	SOYBEAN OIL AND ITS FRACTIONS, ONCE‐REFINED (SUBJECT TO ALKALAI OR CAUSTIC WASH BUT NOT BLEACHED OR DEODORIZED), NOT CHEMICALLY MODIFIED	Once‐refined oil
1507.90.4040	SOYBEAN OIL AND ITS FRACTIONS, FULLY REFINED, WASHED, BLEACHED OR DEODORIZED BUT NOT CHEMICALLY MODIFIED, NESOI	Fully refined oil
1517.10.0000	MARGARINE, EXCLUDING LIQUID MARGARINE	Margarine
1517.90.9025	SOYBEAN OIL, WHOLLY HYDROGENATED, NESOI	Wholly hydrogenated oil
2103.90.9020	MAYONNAISE	Mayonnaise
2103.90.9040	SALAD DRESSINGS, NESOI	Salad dressings
2106.90.2400	BUTTER SUBSTITUTES CONTAINING OVER 10% BY WEIGHT OF MILK SOLIDS, CONTAINING OVER 45% BUTTERFAT, SEE ADDITIONAL U. S. NOTE 14 ‐ CHAP. 4 & PROVISIONAL	Butter substitutes
2106.90.2600	BUTTER SUBSTITUTES CONTAINING OVER 10% BY WEIGHT OF MILK SOLIDS, CONTAINING OVER 45% BUTTERFAT, NESOI	Butter substitutes
2106.90.2800	BUTTER SUBSTITUTES, IN LIQUID OR SOLID STATE, CONTAIN GT 15% BY WEIGHT OF BUTTER OR OTHER FATS OR OILS DERIVED FROM MILK, GT 10% MILK SOLIDS, NESOI	Butter substitutes
2106.90.3600	BUTTER SUBSTITUTES WHETHER IN LIQUID OR SOLID STATE, NESOI, CONTAINING OVER 45 PERCENT BUTTERFAT, NESOI	Butter substitutes
2106.90.3800	BUTTER SUBSTITUTES, WHETHER IN LIQUID OR SOLID STATE, CONTAINING OVER 15% BY WEIGHT OF BUTTER OR OTHER FATS OR OILS DERIVED FROM MILK, NESOI	Butter substitutes
2302.50.0000	BRAN, SHARPS (MIDDLINGS) AND OTHER RESIDUES, WHETHER OR NOT IN PELLETS, DERIVED FROM SIFTING, MILLING OR OTHER WORKINGS OF LEGUMINOUS PLANTS	Brans, midds, residues
2304.00.0000	SOYBEAN OILCAKE AND OTHER SOLID RESIDUES RESULTING FROM THE EXTRACTION OF SOYBEAN OIL, WHETHER OR NOT GROUND OR IN THE FORM OF PELLETS	Soy oilcake
2923.20.2000	LECITHINS AND OTHER PHOSPHOAMINOLIPIDS, NESOI	Lecithins

**TABLE 2:** Top 10 exporters in 2019 of soy to United States with products, quantities (MT) and FAD status^a^, ^b^

		Products											
Country	Total	Non‐organic soybeans	Organic soybeans	Soy oilcake	Soy flours and meals	Soy oil	Bran, midds, residues	Lecithins	Mayonnaise	Salad dressings	Butter and margarine	Soybean seeds	FAD present in country
Canada	546,467	75,846	13,991	190,999	2809	163,888	41,152	4697	10,722	3222	7978	31,162	No
India	397,858	8675	80,681	304,772	1849	615	0.0	1265	0.0	0.8	0.5	0.0	Yes
Argentina	122,116	0.0	88,744	14,815	0.0	1150	16,652	755	0.0	0.0	0.0	0.0	Yes
Ukraine	40,293	0.0	40,143	0.0	0.0	0.0	0.0	68	82	0.0	0.0	0.0	Yes
Turkey	23,348	0.0	456	21,973	0.0	0.0	0.0	0.0	0.0	902	17	0.0	Yes
Russia	21,997	0.0	20,661	0.0	0.0	0.0	0.0	1064	266	0.0	6.2	0.0	Yes
Mexico	20,833	3.2	2180	0.0	256	3498	0.0	0.0	686	11,258	2952	0.0	Yes
Kazakhstan	13,337	0.0	13,337	0.0	0.0	0.0	0.0	0.0	0.0	0.0	0.0	0.0	No
China	9038	1481	137	4449	79	6.9	2681	137	0.0	18	19	30	Yes
Moldova	5986	0.0	5986	0.0	0.0	0.0	0.0	0.0	0.0	0.0	0.0	0.0	Yes
Others^c^	32,816	345	4120	463	885	564	2736	7852	2538	2316	10,997	0.0	Yes
Grand total	1,234,089	86,352	270,437	537,470	5878	169,721	63,221	15,838	14,294	17,717	21,969	31,192	NA

^a^ Countries, products and quantities (MT) were obtained from the United States International Trade and Tariff Database.

^b^ Foreign animal disease status was determined based on presence of African swine fever virus, classical swine fever virus, foot and mouth disease and/or pseudorabies virus in a country during 2019 as reported by the OIE WAHIS Disease Time Chart database.

^c^ Countries included in others: Afghanistan, Australia, Austria, Bangladesh, Barbados, Belarus, Belgium, Brazil, Chile, Colombia, Costa Rica, Croatia, Denmark, Dominican Republic, Ecuador, El Salvador, Ethiopia, France, Germany, Ghana, Greece, Guatemala, Guyana, Haiti, Hungary, Indonesia, Ireland, Israel, Italy, Jamaica, Japan, Jordan, Lithuania, Malaysia, Morocco, Netherlands, Nigeria, North Macedonia, Norway, Peru, Philippines, Poland, Portugal, Serbia, Slovenia, South Korea, Spain, Sweden, Switzerland, Syria, Taiwan, Thailand, Togo, Trinidad and Tobago, United Kingdom, Uruguay and Vietnam.

## 3 RESULTS

### 3.1 Annual soy imports

In 2019, soy products were imported into the United States from 65 different countries. Canada (546,467 metric tonnes, MT), India (397,858 MT) and Argentina (122,116 MT) contributed the most to soy being imported into the United States in 2019 (Table 2). Overall, soy oilcake (537,470 MT) was imported in the largest quantities followed by organic soybeans (270,437 MT) and soy oil (169,721 MT). Soy flour and meal (5878 MT) was the least commonly imported ingredient with mayonnaise (14,295 MT) and lecithins (15,838 MT) following. Eight countries (Argentina, China, India, Mexico, Moldova, Russia, Turkey and Ukraine) within the top 10 exporters of soy to the United States and several countries outside the top 10 had reported FAD cases in 2019. The products these top FAD‐positive countries exported the most to the United States were soy oilcake (346,065 MT), organic soybeans (240,698 MT) and bran, midds and residues (20,594 MT).

The top 10 countries exporting soy products to the United States in 2020 were very similar to the list from 2019. Primary changes were Togo and the Netherlands overtaking Moldova and Kazakhstan (Table 3). Canada (481,497 MT), India (430,621 MT) and Argentina (79,471 MT) were still the top three countries soy products were sourced from in 2020. Soy oilcake (582,273 MT) was imported in the largest quantity through 2020 followed by organic soybeans (270,194 MT) and soy oils (134,436 MT). China, India, Russia and Ukraine were all reported as FAD positive and in the top 10 soy exporters to the United States in 2020. Combined, all FAD‐positive countries in 2020 primarily exported soy oilcake (387,944 MT), organic soybeans (159,041 MT) and lecithins (5000 MT) to the United States.

**TABLE 3:** Top 10 exporters in 2020 of soy to United States with products, quantities (MT) and FAD status^a^, ^b^

		Products											
Country	Total	Non‐organic soybeans	Organic soybeans	Soy oilcake	Soy flours and meals	Soy oil	Bran, midds, residues	Lecithins	Mayonnaise	Salad dressings	Butter and margarine	Soybean seeds	FAD present in country
Canada	481,497	69,987	19,064	152,128	3619	123,502	42,913	3835	11,988	1654	7412	45,393	No
India	430,621	4.1	38,108	387,269	1748	2155	0.0	1337	0.0	0.0	0.8	0.0	Yes
Argentina	79,471	160	65,898	7546	0.0	227	5139	501	0.0	0.0	0.8	0.0	No
Russia	65,666	0.0	64,478	0.0	0.0	0.0	0.0	818	364	0.0	7.2	0.0	Yes
Ukraine	56,392	0.0	56,093	0.0	0.0	0.0	0.0	186	113	0.0	0.0	0.0	Yes
Turkey	40,518	0.0	0.0	34,276	0.0	3906	24	0.0	788	1513	12	0.0	No
Mexico	27,965	18	7518	0.0	405	2952	0.0	0.0	814	13,661	2598	0.0	No
Togo	11,394	318	11,076	0.0	0.0	0.0	0.0	0.0	0.0	0.0	0.0	0.0	No
China	3348	1208	153	468	33	0.0	1269	186	10	22	0.0	0.0	Yes
Netherlands	3222	0.0	0.0	0.0	0.0	18	12	3162	30	0.3	0.2	0.0	No
Others^c^	34,419	1669	7807	588	885	1677	1418	4427	3205	2326	10,418	0.0	Yes
Grand total	1,234,513	73,363	270,194	582,273	6690	134,436	50,775	14,451	17,312	19,176	20,449	45,393	NA

^a^ Countries, products and quantities (MT) were obtained from the United States International Trade and Tariff Database.

^b^ Foreign animal disease status was determined based on prevalence of African swine fever, classical swine fever, foot and mouth disease and/or pseudorabies in a country during 2020 as reported by the OIE WAHIS Disease Time Chart database.

^c^ Countries included in others: Afghanistan, Australia, Austria, Belgium, Benin, Brazil, Chile, Colombia, Costa Rica, Croatia, Czech Republic, Denmark, Dominican Republic, Ecuador, Egypt, Ethiopia, France, Germany, Greece, Guatemala, Guyana, Haiti, Hungary, Indonesia, Iran, Ireland, Israel, Italy, Jamaica, Japan, Jordan, Kenya, Lithuania, Malaysia, Morocco, Nigeria, North Macedonia, Norway, Paraguay, Peru, Philippines, Poland, Portugal, Rwanda, Senegal, Serbia, Singapore, Slovenia, South Korea, Spain, Sweden, Switzerland, Syria, Taiwan, Thailand, Trinidad and Tobago, United Arab Emirates, United Kingdom, Uruguay, Venezuela and Vietnam.

### 3.2 Ports of entry

Soy products sourced from the top 10 exporters to the United States in 2019 most commonly entered into the United States at 13 different ports. The port that handled the largest quantity of soy products from a country was defined as the primary POE for that country. The secondary and tertiary POE for a country handled the second and third largest quantities, respectively, of soy products being imported to the United States from that country. The top three countries in 2019 that the United States imported soy products from were Canada, Argentina and India. Canada primarily used Detroit, MI (204,622 MT) to send soy products to the United States (Table 4). Baltimore, MD handled the next largest volume for the primary POE and was utilized by Argentina (51,507 MT) and India (132,973 MT). Both China and Turkey sent most of their soy exports to the United States through San Francisco, CA (4357 MT and 11,665 MT, respectively). New Orleans, LA was the most common primary POE for Kazakhstan (11,062 MT), Moldova (5986 MT), Russia (14,860 MT) and Ukraine (26,218 MT). Laredo, TX was the primary POE for products coming from Mexico (17,013 MT). For secondary POE, Buffalo, NY handled the highest volume (126,913 MT) of soy imports from top 10 countries. San Francisco, CA was the secondary POE for India, which sent 110,806 MT of soy product through this port in 2019. The most common secondary POE among the top 10 soy exporters to the United States was Charlotte, NC with 57,788 MT coming through this port from Argentina, Kazakhstan, Russia or Ukraine.

**TABLE 4:** Top 10 exporters of soy and primary United States ports of entry with quantity (MT) in 2019 and 2020^a^

Country	Primary port		Second port		Third port	
2019						
Argentina	Baltimore, MD	51,507	Charlotte, NC	36,588	San Francisco, CA	18,544
Canada	Detroit, MI	204,622	Buffalo, NY	126,913	Ogdensburg, NY	122,478
China	San Francisco, CA	4357	Los Angeles, CA	2464	Seattle, WA	880
India	Baltimore, MD	132,973	San Francisco, CA	110,806	Seattle, WA	67,781
Kazakhstan	New Orleans, LA	11,062	Charlotte, NC	2275	NA	NA
Mexico	Laredo, TX	17,013	San Diego, CA	2454	El Paso, TX	971
Moldova	New Orleans, LA	5986	NA	NA	NA	NA
Russia	New Orleans, LA	14,860	Charlotte, NC	5000	Ogdensburg, NY	801
Turkey	San Francisco, CA	11,665	New Orleans, LA	10,000	New York, NY	879
Ukraine	New Orleans, LA	26,218	Charlotte, NC	13,925	New York, NY	88
2020						
Argentina	Charlotte, NC	35,541	Houston‐Galveston, TX	12,953	San Juan, PR	12,685
Canada	Detroit, MI	169,946	Buffalo, NY	146,466	Ogdensburg, NY	76,879
China	Los Angeles, CA	1665	New York, NY	561	Seattle, WA	475
India	Baltimore, MD	147,110	San Francisco, CA	126,782	Seattle, WA	52,256
Mexico	Laredo, TX	18,809	Nogales, AZ	4261	San Diego, CA	3827
Netherlands	Chicago, IL	1729	New York, NY	454	Los Angeles, CA	387
Russia	New Orleans, LA	63,462	Seattle, WA	814	New York, NY	613
Togo	San Francisco, CA	6120	Norfolk, VA	4046	Philadelphia, PA	678
Turkey	New Orleans, LA	24,214	San Francisco, CA	13,422	New York, NY	1414
Ukraine	New Orleans, LA	51,093	Charlotte, NC	5000	New York, NY	164

Abbreviation: NA, not applicable.

^a^ Countries of origin, ports of entry and quantities (MT) of soy imports were obtained from the United States International Trade and Tariff Database.

Comparing the POE for the top 10 soy exporters to the United States in 2019–2020 reveals several changes across the year. Argentina, China and Turkey all had their secondary POE from 2019 shift to their primary port in 2020 (Charlotte, NC; Los Angeles, CA; and New Orleans, LA, respectively). Argentina also utilized Houston‐Galveston, TX (12,953 MT) and San Juan, PR (12,685 MT) for a large quantity of soy exported to the United States. Mexico utilized Nogales, AZ (4261 MT) secondary to Laredo, TX (18,809 MT) in 2020, bumping San Diego, CA to third with 3827 MT. Chicago, IL handled the highest quantity of soy products being imported from the Netherlands at 1729 MT. Russia sent its second highest quantity of soy through Seattle, WA (814 MT) in 2020 as opposed to Charlotte, NC in 2019. San Francisco, CA handled the highest quantity of imports from Togo (6120 MT) followed closely by Norfolk, VA (4046 MT).

The top five POEs by volume were evaluated by amount of each product type imported in 2019. Detroit, MI handled the highest volume of soy products imported to the United States during this year (215,755 MT), followed by Baltimore, MD (185,578 MT), San Francisco, CA (146,279 MT), Ogdensburg, NY (144,057 MT) and Buffalo, NY (127,279 MT) (Table 5). Between these ports, Detroit brought in the largest quantity of non‐organic soybeans (51,183 MT) and Baltimore handled the largest amount of organic soybeans (60,191 MT). Most of the soy oilcake entered the United States through either Baltimore (119,461 MT) or San Francisco (116,264 MT). Ogdensburg, NY handled the most soy flour and meal (1631 MT) and soy oil (80,444 MT) of these five ports. Brans, middlings and other residues were primarily imported to the United States by Buffalo, NY (19,732 MT) and Detroit (11,985 MT). In 2020, the top five POEs were Detroit, MI (190,311 MT), New Orleans, LA (183,786 MT), Baltimore, MD (162,445 MT), San Francisco, CA (151,123 MT) and Buffalo, NY (146,794 MT). Most of the non‐organic soybeans imported through these five ports entered the United States through Detroit (53,050 MT). New Orleans lead the top five POEs in imports of organic soybeans with 136,709 MT. Soy oilcake was imported in large quantities through all five ports, with Baltimore and San Francisco handling the most (146,912 and 126,820 MT, respectively). Detroit also processed the most soy flour and meal (1781 MT) and soy oil (79,805 MT) out of the top five POEs in 2020. Bran, middlings and residues were only imported through Buffalo and Detroit out of these five ports as well (20,045 and 8762 MT, respectively).

**TABLE 5:** Top five ports of entry and quantity (MT) of associated products in 2019 and 2020^a^

	Ports of entry for 2019				
Product	Detroit, MI	Baltimore, MD	San Francisco, CA	Ogdensburg, NY	Buffalo, NY
Non‐organic soybeans	51,183	5679	882	5310	5310
Organic soybeans	594	60,191	17,345	19,489	19,489
Soy oilcake	68,395	119,461	116,264	35,899	71,799
Soy flour and meals	282	0.6	223	1631	1.1
Soy oils	66,115	181	1045	80,444	5754
Bran, midds, residues	11,985	0.0	9570	149	19,732
Lecithins	4713	15	934	18	6.9
Mayonnaise	4990	9.5	4.4	18	5728
Salad dressings	1928	41	6.8	49	1110
Margarine	5572	0.0	4.9	1050	630
Soybean seeds	0.0	0.0	0.0	0.0	43
Total	215,755	185,578	146,279	144,057	127,279

	Ports of Entry for 2020				
Product	Detroit, MI	New Orleans, LA	Baltimore, MD	San Francisco, CA	Buffalo, NY
Non‐organic soybeans	53,050	0.0	733	1.8	9348
Organic soybeans	2600	136,709	13,552	18,702	2637
Soy oilcake	26,706	46,904	146,912	126,820	104,278
Soy flour and meals	1781	0.0	0.0	1.7	120
Soy oils	79,805	0.0	228	4966	4503
Bran, midds, residues	8762	0.0	0.0	0.0	20,045
Lecithins	3894	172	67	595	43
Mayonnaise	7982	0.0	785	22	4002
Salad dressings	612	0.0	169	8.6	718
Margarine	4129	0.0	0.0	5.3	752
Soybean seeds	989	0.0	0.0	0.0	348
Total	190,311	183,786	162,445	151,123	146,794

^a^ Product types, ports of entry and quantities (MT) of soy imports were obtained from the United States International Trade and Tariff Database.

### 3.3 Imports from countries with FAD

**FIGURE 1:**
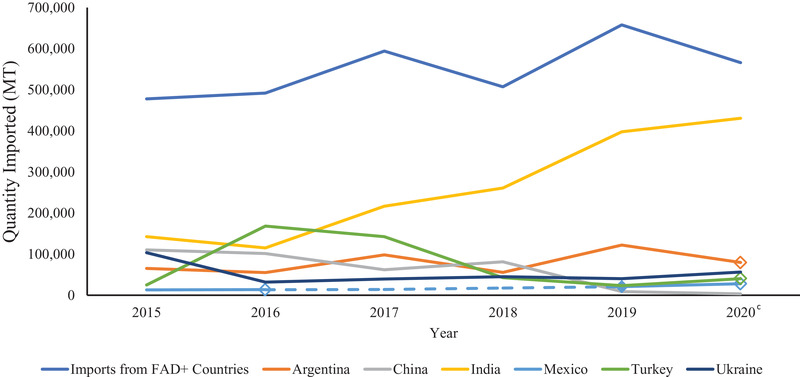
Imports from countries with foreign animal disease from 2015 to 2020 and the top six exporters of soy to the United States by quantity (MT)^a,b^ *Note*: Dashed lines indicate years that a country did not have FAD status reported or there were no positive cases. Diamond markers are the single year that a country had reported FAD cases. Open diamond markers indicate the beginning of years that the country is FAD negative or did not have reported cases. ^a^Countries of origin and quantities (MT) of soy imports were obtained from the United States International Trade and Tariff Database. ^b^Foreign animal disease status was determined based on presence of African swine fever virus, classical swine fever virus, foot and mouth disease and/or pseudorabies in a country during each year as reported by the OIE WAHIS Quantitative Data database. ^c^No differentiation provided in figure between countries reporting no FAD cases and countries with no FAD data for 2020 in the OIE WAHIS Quantitative Database.

From 2015 to 2020, imports from reported FAD‐positive countries increased from 477,806 to 566,318 MT (Figure 1). This increase was not consistent from year to year during this time period with large increases in imports in 2017 and 2019 followed by a decrease in 2018 and 2020. The year that had the greatest quantity of soy imports from countries with FAD cases was 2019 with 657,812 MT. India, China and Ukraine were the top exporters of soy products to the United States that had a consistently positive FAD status over these 6 years.

**FIGURE 2:**
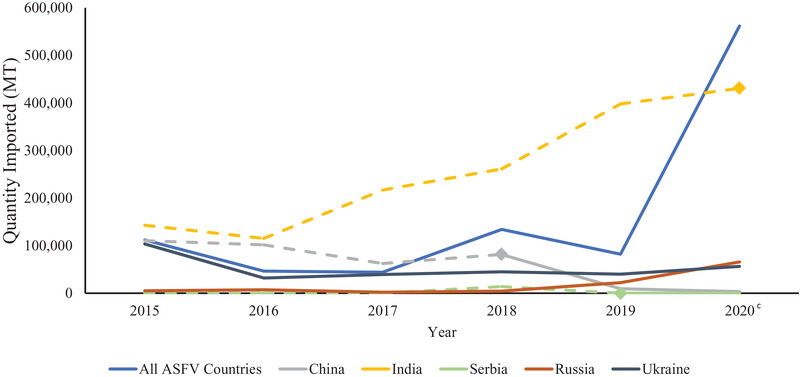
Imports from countries with African swine fever virus from 2015 to 2020 and the top five exporters of soy to the United States by quantity (MT)^a,b^ *Note*: Dashed lines indicate years that a country did not have ASFV status reported or there were no positive cases. Diamond markers are the year that a country began to have reported ASFV cases. ^a^Countries of origin and quantities (MT) of soy imports were obtained from the United States International Trade and Tariff Database. ^b^African swine fever status was determined based on presence of cases in a country during each year as reported by the OIE WAHIS Quantitative Data database. ^c^No differentiation provided in figure between countries reporting no ASFV cases and countries with no ASFV data for 2020 in the OIE WAHIS Quantitative Database.

From 2015 to 2020, imports from ASFV‐positive countries have varied from 44,047 to 561,583 MT (Figure 2). Ukraine was the largest contributor of imports from ASFV‐positive countries from 2015 to 2018 when it was overcome by China. China exported more soy products to the United States than Ukraine from 2015 through 2018, but did not have cases of ASFV reported until 2018. Imports from China decreased in 2019 and 2020, causing Ukraine and India to be the largest contributors to U.S. imports from ASFV‐positive countries in these years, respectively. In 2020, India reported positive cases of ASFV, leading to a drastic increase in imports with ASFV risk. From 2015 to 2020, Russia and Ukraine were the only countries that were ASFV positive every year. Russia only fluctuated between 2083 and 7241 MT from 2015 to 2018 and then increased to 21,997 MT in 2019 and increased even more in 2020 to 65,666 MT. Ukraine exported between 31,916 and 103,559 MT to the United States from 2015 to 2020 with the peak in 2015.

**FIGURE 3:**
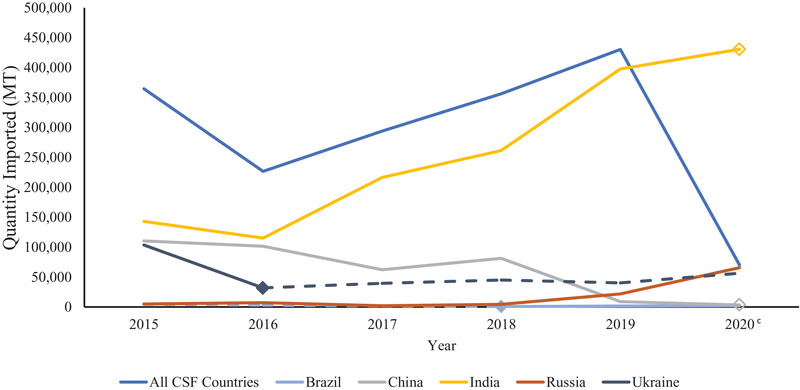
Imports from countries with classical swine fever virus from 2015 to 2020 and the top five exporters of soy to the United States by quantity (MT)^a,b^ *Note*: Dashed lines indicate years that a country did not have CSFV status reported or there were no positive cases. Diamond markers are the year that a country began to have reported CSFV cases. Open diamond markers indicate the beginning of years that the country is CSFV negative or did not have reported cases. ^a^Countries of origin and quantities (MT) of soy imports were obtained from the United States International Trade and Tariff Database. ^b^Classical swine fever status was determined based on presence of cases in a country during each year as reported by the OIE WAHIS Quantitative Data database. ^c^No differentiation provided in figure between countries reporting no CSFV cases and countries with no CSFV data for 2020 in the OIE WAHIS Quantitative Database.

Soy imports from countries with CSFV outbreaks dropped slightly from 2015 to 2016 (364,409 to 226,642 MT) followed by a steady increase from 2016 to 2019 with a sharp decrease in 2020 (Figure 3). Ukraine was CSFV positive in 2015, but not in 2016, resulting in the drop in imports from CSFV‐positive countries between these years. India was CSFV positive from 2015 to 2019 and the increase of imports with CSFV risk from 2016 to 2019 matches the increased quantity of exports to the United States during this time period. Although India had its largest year for soy exports to the United States in 2020, it did not have reported cases of CSFV in this year which caused the sharp drop in imports with CSFV risk.

**FIGURE 4:**
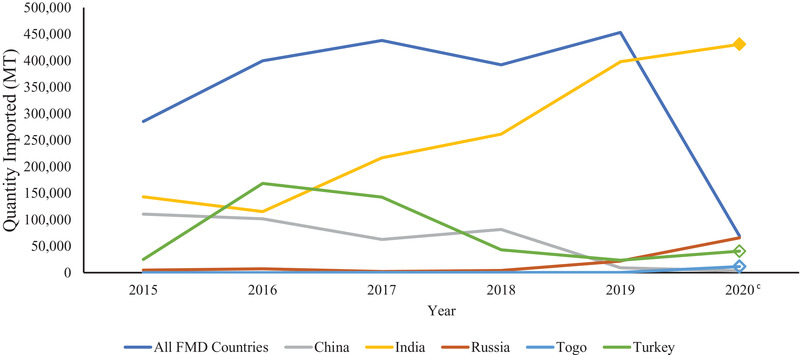
Imports from countries with foot and mouth disease from 2015 to 2020 and the top five exporters of soy to the United States by quantity (MT)^a,b^ *Note*: Open diamond markers indicate the beginning of years that the country is FMDV negative or did not have reported cases. ^a^Countries of origin and quantities (MT) of soy imports were obtained from the United States International Trade and Tariff Database. ^b^Foot and mouth disease status was determined based on presence of cases in a country during each year as reported by the OIE WAHIS Quantitative Data database. ^c^No differentiation provided in figure between countries reporting no FMDV cases and countries with no FMDV data for 2020 in the OIE WAHIS Quantitative Database.

Imports from FMDV‐positive countries demonstrated an increase from 285,146 to 453,149 MT from 2015 to 2019 followed by a sharp decrease to 69,019 MT in 2020 (Figure 4). Over this period, China and Russia remained FMDV positive each year and imports from China decreased over this time and Russia gradually increased. India was FMDV positive from 2015 to 2019 and was the largest contributor to imports with FMD risk from 2017 to 2019. India did not have FMD cases reported in 2020, leading to Russia becoming the largest contributor in this year.

**FIGURE 5:**
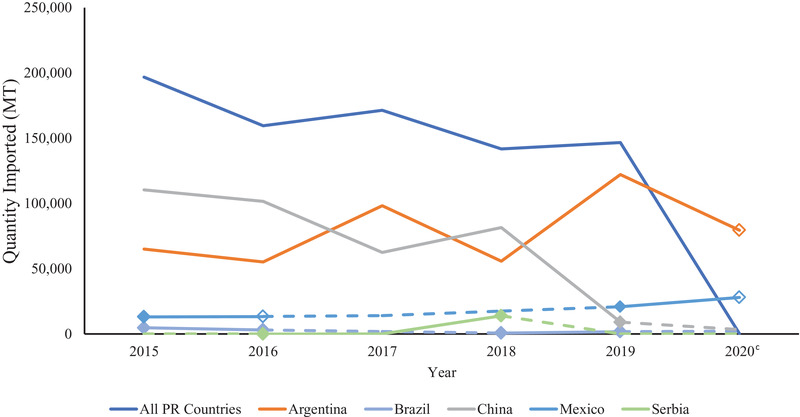
Imports from countries with pseudorabies from 2015 to 2020 and the top five exporters of soy to the United States by quantity (MT)^a,b^ *Note*: Dashed lines indicate years that a country did not have pseudorabies status reported or there were no positive cases. Diamond markers are the year that a country began to have reported pseudorabies cases. Open diamond markers indicate the beginning of years that the country is pseudorabies negative or did not have reported cases. ^a^Countries of origin and quantities (MT) of soy imports were obtained from the United States International Trade and Tariff Database. ^b^Pseudorabies status was determined based on presence of Aujeszky's disease cases in a country during each year as reported by the OIE WAHIS Quantitative Data database. ^c^No differentiation provided in figure between countries reporting no pseudorabies cases and countries with no pseudorabies data for 2020 in the OIE WAHIS Quantitative Database.

Imports from pseudorabies‐positive countries demonstrated a gradual decrease from 196,771 to 146,662 MT from 2015 to 2019, followed by a sharp decline to 203 MT in 2020 (Figure 5). China and Argentina alternated as the largest contributor to imports from pseudorabies‐positive countries from 2016 to 2019. Both China and Argentina demonstrated variability in soy exports to the United States from 2015 to 2020 and appear to have an inverse relationship where an increase of imports from one correlated to a decrease in imports from the other. France was the only country that reported positive cases in 2020, leading to the sharp drop in imports from pseudorabies‐positive countries in this year.

## 4 DISCUSSION

The soy‐based HTS codes used were selected because of the product's potential to be included in swine diets in their imported state with minimal processing or their potential to be used as a by‐product from the food industry. Several product groups, such as salad dressings, are not very likely to end up in a large volume of swine diets and therefore pose less disease risk. Products such as lecithins and butter substitutes also are not likely to be used in swine diets in their current forms but their inclusion in by‐products fed to livestock gives them a greater chance of being added to swine diets. Thermal processing of feedstuffs, such as bakery by‐products, may kill or inactivate pathogens in the original ingredients, but recontamination of the by‐product post‐processing may allow viable virus to survive. In addition, this paper identified soy imports from FAD‐positive countries. These countries were determined based on reported cases in the OIE WAHIS database and could change as more information becomes available. For example, very few countries had data reported for pseudorabies in 2020 when the database was queried for this publication. The lack of data caused several countries to be considered ‘negative’ for pseudorabies in 2020 even though they had not reported being free of pseudorabies.

Previous research has demonstrated the ability of soybean meal to serve as a vector for active virus, and as a result is a product of interest when evaluating FAD introduction risk (Dee et al., 2018; Stoian et al., 2020). The current evaluation found organic soybean meal to be a very small portion of the imports into the United States, with 6690 MT of organic and non‐organic soybean meal being imported out of 1,234,513 MT of total soy in 2020. Less than a third of that soybean meal (2163 MT) is sourced from countries with FAD cases which could be viewed as a low probability of disease introduction, but the severity and economic impact of disease introduction is still high. With an infectious dose of 10^6.8^ TCID_50_ for ASFV, 300 TCID_50_ for FMDV and 10^4.2^ TCID_50_ for CSFV, even a small amount of virus entering the country can lead to infection of U.S. swine herds (Alexandersen et al., 2002; Cowan et al., 2015; Niederwerder et al., 2019). Smaller amounts of virus in feed can still lead to infection due to pigs eating throughout the day and accumulating virus at or above that infectious level.

Although organic soybean meal may not be a large contributor to U.S. soy imports, soy oilcake is imported in large quantities. Soy oilcake is the by‐product of compressing soybeans to extract the soy oil. This oilcake can then be ground into soybean meal and included in swine diets. Soy oilcake made up 47% of soy imports in 2020, with 67% of the soy oilcake being imported from reported FAD‐positive countries. The largest contributor, overall and of reported FAD‐positive countries, of soy oilcake being imported into the United States in 2020 was India (ASFV‐positive) with 387,269 MT. The current HTS codes do not differentiate between organic and non‐organic soy oilcake; therefore, it was not possible to quantify the amount of this product that is organic using the USITC DataWeb. Dee et al. (2018) have shown that soy oilcake is able to harbour viable, infectious virus through an international shipping model. The need to grind the soy oilcake down to soybean meal before use adds another point of concern. Viral contamination of feed processing facilities has been shown to occur after the handling of contaminated feed ingredients and decontamination can be difficult (Elijah et al., 2020; Huss et al., 2017). Although these studies did not address the grinding step, the production of dust during this stage of processing does provide potential for one contaminated batch of soy oilcake to infect subsequent batches by contaminating the equipment and environment in the manufacturing facility. This could allow one shipment of contaminated oilcake to inoculate multiple shipments of formerly uncontaminated oilcake or other ingredients that are processed through the same facility. Organic soybeans also were a large portion of soy imports contributing 22% of the total soy imports from 2020. Being able to understand how these soybeans are being used, whether for human consumption, livestock feed or oil extraction, will be beneficial to understand the risk of these beans introducing disease to U.S. swine herds. Although they are imported at about 4% of the total volume of soy products, other by‐products such as bran, middlings and residues are important to keep in mind when considering soy imports because of the possibility of these products being used as a fibre source in swine diets.

POE can also be an important factor in disease risk of feed ingredients due to the variation of time required for transport. Routes that require significant land or air transport prior to reaching a seaport for trans‐Atlantic or trans‐Pacific shipping will have different environmental challenges to viruses and therefore require different holding times. Although many imports are entering ports on the coasts, such as San Francisco or Baltimore, a fair number of imports from reported FAD‐positive countries are entering through southern ports such as New Orleans and Charlotte, NC. These latter two ports have not been included in previous trans‐boundary shipping models and the use of water transportation to get ingredients to their final destination may have a different effect on virus survivability (Dee et al., 2018; Dee et al., 2016; Stoian et al., 2020).

From 2015 to 2019, soy imports from reported FAD‐positive countries have increased. Among the top six countries with FAD outbreaks, only China, India and Ukraine had cases every year (Figure 1). Imports from China sharply declined in 2019, but there are factors outside of the FAD status of the country that could have contributed to this. Several tariffs were placed against China in 2018 and 2019 (United States Trade Representative, Section 301‐ China, List 1–4) and these actions likely contributed to the sharp decline due to the inclusion of many soy products in the tariffs included on List 3. India was the largest contributor to the increase of imports from reported FAD‐positive countries from 2017 to 2020. The increase in soy imports from countries with FAD cases from 2018 to 2019 follows a similar trend to the increase in imports from India in this time.

ASFV and surrogates for FMDV and pseudorabies have been demonstrated to survive transboundary shipping in soy ingredients, such as soybean meal and soy oilcake (Dee et al., 2018). Pseudorabies has been eradicated from U.S. domestic swine herds, but is currently endemic in feral swine populations (Brown et al., 2019). This eradication was possible, in part, because of the availability of a vaccine for pseudorabies, and domestic swine herds can be vaccinated if pseudorabies is re‐introduced (Haagmans et al., 1999). Classical swine fever virus has been eradicated from the United States since the 1970s, but this virus still exists in portions of Central and South America, Asia and Africa (Brown & Bevins, 2018b). Vaccines against CSFV currently exist and can be utilized if CSFV is introduced into U.S. swine populations (Beer et al., 2007). FMDV introduction to U.S. herds is particularly detrimental because of the large number of species that can be affected (Knight‐Jones & Rushton, 2013). A vaccine for FMDV exists, but vaccination plans will likely involve movement restrictions within a certain area surrounding an outbreak (Knight‐Jones & Rushton, 2013). African swine fever is endemic in portions of Africa and Europe and currently has no vaccine (Brown & Bevins, 2018a). Introduction of ASFV to U.S. swine herds would have a large, detrimental impact because of the lack of vaccination possibility and likelihood of trade restrictions.

Imports from ASFV‐positive countries decreased from 2015 until a spike in 2018. The addition of China to the list of positive countries in 2018 was most likely the reason for this increase and positive ASFV cases in India led to an even more drastic increase in 2020. Soy products sourced from countries with CSFV outbreaks saw a decrease from 2015 to 2016 and a steady rise from 2016 to 2019 with a sharp decrease in 2020. Once again, India contributed to the rise in imports from CSFV‐positive countries across this span, and the lack of reported CSFV cases in the country in 2020 led to the sharp decline of soy products imported from CSFV‐risk countries. Soy imports sourced from countries with FMDV‐positive status also experienced an increase from 2015 to 2019 followed by a decline in 2020. A large portion of that can be correlated to imports from India from 2016 to 2019, as that was the country that contributed the most to soy imports in this category. The decrease in imports from FMDV‐positive countries in 2020 is due to India no longer having reported FMDV cases, leaving Russia to be the largest contributor of imports with FMDV risk for this year. Pseudorabies‐positive countries are the only FAD category in this investigation that decreased exports to the United States from 2015 to 2019. For the first three diseases, India was a large driver in import trends. In contrast, China and Argentina contributed the most to imports with pseudorabies risk. Argentina was one of the largest exporters of soy products to the United States in 2019 and 2020, so its inclusion in pseudorabies‐positive countries had a large impact. Prior to 2017, China exported more soy products to the United States than Argentina, increasing the amount of imports from countries experiencing pseudorabies outbreaks. In 2017, Argentina overtook China for the top pseudorabies‐positive country and these two alternated the top position each year through 2019. In 2020, France was the only country with pseudorabies reported, so quantities of soy sourced from pseudorabies‐positive countries dropped drastically. The OIE WAHIS database is updated with each report put out by the World Organization for Animal Health, whether that is a quarterly, 6‐month or annual report. Due to this fact, the FAD status of countries within this timeframe may change as countries that had no data for a year report positive cases or a negative status. As a result, numbers for 2020 will likely change with the release of more information.

Overall, imports from reported FAD‐positive countries contributed about 53% of the total soy imports in 2019 with India, Argentina and Turkey being the largest individual contributors within this group. In 2020, approximately 46% of the total soy import was sourced from reported FAD‐positive countries. This high percentage is primarily due to reports of FAD in India and Argentina. Both of these were among the top three exporters of soy products to the United States for each year. The trend of imports from ASFV‐positive countries is a prime example of the impact of even one major soy exporter experiencing a disease outbreak. India breaking with ASFV in 2020 lead to a drastic increase in imports sourced from ASFV‐positive countries. It also should be noted that this information does not take into account that the products imported from FAD‐negative countries may have been imported from somewhere else previously. Similar to a statement expressed by Patterson et al. (2021), the interconnectedness of the global economy makes it difficult to trace the original source of products in some cases. A deeper look into where a region's products are being sourced from would be beneficial in understanding the disease risk of the product more objectively. The end use is also an important consideration because a product that is used exclusively for human or industrial consumption also has a low disease risk, even if it is contaminated, because of its removal from interaction with swine herds.

## 5 CONCLUSION

Understanding the sources and intended uses of products being imported to the United States is vital to determine the risk of FAD disease introduction. Quantifying the amount and country of origin for imports into the United States is beneficial to start digging deeper into the biosecurity of feed across the country. Although this quantification is beneficial, it should not be taken as a defining declaration of the risk of FAD introduction without a holistic view of the storage, transport and usage of imported soy products. Being able to monitor FAD disease outbreaks and imports from countries could be useful for evaluating the risk of FAD introduction into U.S. swine herds more readily.

## CONFLICT OF INTEREST

The authors declare no conflict of interest.

## ETHICS STATEMENT

The authors confirm that the ethical policies of the journal, as noted on the journal's author guidelines page, have been adhered to. No ethical approval was required as this is a review article with no original research data.

## Data Availability

The data that support the findings of this study are available from corresponding author upon reasonable request.
